# A single reaction-diffusion equation for the multifarious eruptions of urticaria

**DOI:** 10.1371/journal.pcbi.1007590

**Published:** 2020-01-15

**Authors:** Sungrim Seirin-Lee, Yuhki Yanase, Shunsuke Takahagi, Michihiro Hide

**Affiliations:** 1 Department of Mathematics, School of Science, Hiroshima University, Kagamiyama, Higashi-Hiroshima, Japan; 2 Department of Mathematical and Life Sciences, Graduate School of Integrated Sciences for Life, Hiroshima University, Kagamiyama, Higashi-Hiroshima, Japan; 3 JST PRESTO, 4-1-8 Honcho, Kawaguchi, Saitama, Japan; 4 Department of Dermatology, Graduate School of Biomedical Sciences, Hiroshima University, Kasumi, Minami-ku, Hiroshima, Japan; Mathematical Institute and the Institue for Biology, Leiden, NETHERLANDS

## Abstract

Urticaria is a common skin disorder characterized by the rapid appearance and disappearance of local skin edema and flares with itching. It is characterized by various macroscopic skin eruptions unique to patients and/or subtypes of urticaria with respect to shape, size, color, and/or duration of eruptions. Nevertheless, the mechanism underlying multifarious eruptions in urticaria is largely unknown. The eruptions are believed to be evoked by histamine release from mast cells in the skin. However, the majority of visible characteristics of urticaria cannot be explained by a simple injection of histamine to the skin. To explain the multifarious eruptions of urticaria, we developed a single reaction-diffusion model suggesting the self-activation and self-inhibition regulation of histamine release from mast cells. Using the model, we found that various geometrical shapes of eruptions typically observed in patients can be explained by the model parameters and randomness or strength of the initial stimuli to mast cells. Furthermore, we verified that the wheal-expanding speed of urticaria, which is shown to be much smaller than that of the intradermal injection experimental system may be explained by our model and a simple diffusion equation. Our study suggests that the simple reaction-diffusion dynamics, including the independent self-activating and -inhibitory regulation of histamine release, may account for the essential mechanism underlying the formation of multifarious eruptions in urticaria.

## Author summary

Urticaria is a common skin disease but the mechanism underlying wheal formation is not well understood. Our mathematical model suggests that not only the self-activation of histamine production via mast cells, but also self-inhibition of histamine dynamics plays a critical role in generating the wide-spread wheal patterns observed in urticaria; this has not been previously considered in medicine. The study findings may increase the understanding of the pathogenesis of urticaria and may aid decision-making for appropriate treatments. It may also open an entirely new avenue for mathematical approaches to analyze various skin diseases with geometric eruptions and predict the effectiveness of treatments through *in silico* experiments.

## Introduction

**Fig 1:**
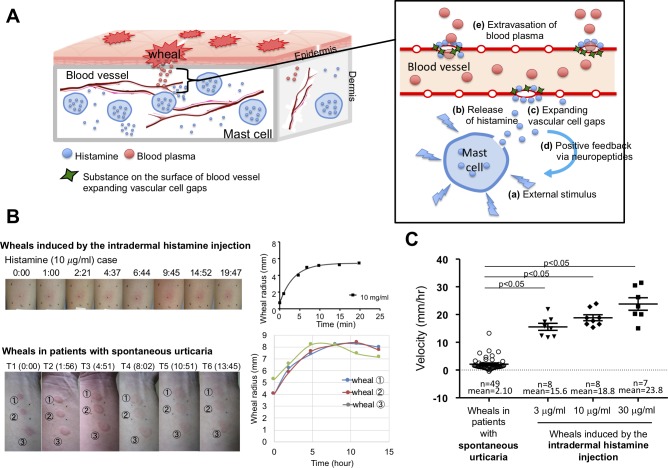
Histamine dynamics and expanding speed of wheals in experiments vs wheals observed in patients with chronic spontaneous urticaria. (A) The process of urticarial development. Dermal mast cells are stimulated and degranulate, releasing mediators including histamine (a, b). Released histamine acts on vascular endothelial cells and sensory neurons to induce the formation of intercellular gaps (c) and the release of neuropeptides which activate mast cells in the vicinity (d). The blood plasma exudates through the gap and wheals develop (e). (B) The upper left panel shows photographic images of a wheal induced by the intradermal injection of histamine and the lower left panel shows wheals observed in a patient with chronic spontaneous urticaria (CSU) over a time course. Right panels show the evolution of wheals in terms of their size, showing that wheals in CSU expand much slower than those induced by a bolus injection of histamine. (C) Comparison of the radial expansion velocities of wheals that developed in patients with CSU and of those induced by a bolus intradermal injection of histamine. The maximum radial expanding velocity of wheals induced by intradermal injections of histamine was calculated as the average velocity during the initial 15 minutes after the injection, and that in CSU was calculated as the fastest of the average velocity during the observational time of two adjacent points. The radial expanding velocity of histamine-induced wheals at the indicated concentrations was at least 7 times greater than that of wheals developed in CSU.

Urticaria is a common skin disorder characterized by the transient and repetitive appearance of eruptions, i.e. wheal and flare response with itching on the skin. It affects about 20% of people (one in 5 people) at some point in their lives and globally about 56/100000 population suffer from urticaria daily [1, 2]. Urticaria is classified as the chronic type when it lasts for 6 weeks or longer, and is further divided into chronic spontaneous urticaria (CSU) and chronic inducible urticaria [3, 4]. Chronic urticaria has a significant impact on quality-of-life due to regular recurrence of disfiguring eruptions with itching, and unknown etiology [1, 5]. Moreover, urticaria may be a symptom of anaphylaxis that seriously affects the patient's life. Urticaria is induced by vasoactive mediators, such as histamine, released from mast cells into the tissue, which then induces dilatation and hyperpermeability of the microvasculature (Fig 1A) [3, 6]. Mast cells release their mediators not only in response to antigens that crosslink the high affinity IgE receptors (FcεRI) on their surface, but also to a variety of stimuli, including neuropeptides, adenosine triphosphate (ATP), anaphylatoxins and chemicals, such as polymyxin B [7–10]. The crucial role of histamine in the pathogenesis of urticaria has well been demonstrated by mast cell degranulation revealed by histological inspections [11]; increase of histamine together with other mast cell-derived mediators, such as tryptase in the tissue fluid of lesional tissue and/or plasma [12, 13], and the marked effects of antihistamines observed in many patients [14, 15]. Moreover, intradermal injection of histamine induces flares and wheals that resemble the eruptions that are observed in urticaria [16].

Nevertheless, the underlying mechanism of mast cell activation in the clinical setting has remained largely unclear. The eruptions of certain types of urticaria may be induced by specific antigens, drugs or physical stimuli, such as mechanical scratch or cold. However, wheals of CSU, the most common subtype of urticaria, develop without apparent stimuli. Moreover, a simple injection of histamine by intradermal injection or electrophoresis in healthy individuals does not result in wheals reminiscent of the various shapes and dynamics observed in CSU [17]. On the other hand, eruptions of urticaria developing in individual patients tend to show a certain pattern of shape and kinetics, such as small dots, round, annular and flower-like appearances with various durations ranging from shorter than an hour to almost a day or even longer [18]. Moreover, the efficacy of antihistamines in urticaria is extremely variable, and not necessarily determined by the clinical severity of urticaria. [3, 19]. Therefore, morphological analysis of such eruptions of urticaria may reveal the mechanism of action of histamine in more detail, especially with respect to its diffusion with kinetics, and offer a clue for more personalized treatment. Nevertheless, current recommendations in the guidelines for urticaria do not distinguish the eruptions of urticaria with respect to the treatment, and the mechanism that induces such multifarious eruptions has never been explored.

Because the detailed underlying molecular mechanism of urticaria is still not clear, we first approached the problem using mathematics in order to find a possible principle of the multifarious eruptions of urticaria and its fundamental mechanism. Medically, skin reactions to mast cell activation are believed to be evoked by the cascaded positive feedback of histamine release from mast cells and actions of sensory nerves in the skin (Fig 1A) [4, 20]. This may be captured by a reaction and diffusion dynamics of histamine. Reaction-diffusion equations are one of a well-known pattern-forming system based on the dynamics of two (or more) biochemicals, each of which often plays a role as an activator and inhibitor [21]. Such pattern-forming systems suggest that self-activation and inhibition play a key role in creating spatial heterogeneity. In fact, mast cells may also play an inhibitory role in the development of skin inflammation. Mast cells release various mediators, including proteases together with histamine. Some of them may either further activate mast cells via protease-activated receptor (PARs) on mast cells, or inhibit mast cell activation by reducing thrombin activity which is enhanced by histamine [22, 23]. The only known medical hypothesis to date regarding the action of mast cells in urticaria is the positive feedback of histamine release, namely, activation dynamics of histamine via mast cells [4, 20]. This is likely to induce the simultaneous increase of histamine over the whole space because mast cells are distributed over the whole skin [11]. Thus, in order to find a mechanism for histamine to create macro-scale’s spatial heterogeneity, we have introduced an unestablished but potential mechanism of inhibition regulatory in histamine dynamics by formulating a conceptional mathematical model suggesting the self-activating and self-inhibitory regulation of histamine release by mast cells. Using the model, we succeeded in regenerating wheal formations in comparison with those observed in real patients and explaining various geometrical shapes of eruptions by model parameters and the spatial randomness or strength of initial stimuli to mast cells. Furthermore, we verified that the wheal expanding speed of urticaria, which is shown to be much smaller than that of the intradermal injection experiment, may be explained by our self-activating and self-inhibitory model and a simple diffusion equation. Our study proposes not only the possible underlying mechanism of urticaria for multifarious eruptions, but also a novel pattern-forming dynamics based on pattern transition and a hidden possibility of single reaction-diffusion equation as a pattern-formation framework.

## Results

### Expanding speed of wheals in experiments vs wheals observed in patients with chronic spontaneous urticaria

To understand difference in the dynamics of urticaria and the dynamics of a simple intradermal injection of histamine, we first evaluated the velocity of wheal expansion in the skin of healthy individuals in response to 20 *μl* of intradermal histamine injections at concentrations of 3, 10, and 30 *μg*/*ml* and compared them with the wheals observed in CSU. The size of the wheals reached a plateau in approximately 15 minutes, and the initial velocity increased in a dose-dependent manner for the injected histamine (mean±SE; 15.6±1.3, 18.8±1.1, 23.8±2.3) (Fig 1B upper panels and Fig 1C). In contrast, wheals that developed in patients with CSU continued to expand over hours at velocities much smaller than those induced by histamine injection (Fig 1B lower panels and Fig 1C). These results indicate that neither pin-point release of histamine followed by its diffusion to the surrounding tissues nor simultaneous release of histamine from whole mast cells in the lesion can explain a full-fledged structure and dynamics of wheals development in CSU. Furthermore, the histamine dynamics in the skin cannot be explained by the cascaded positive feedback alone of histamine release from mast cells.

### Development of mathematical model

**Fig 2:**
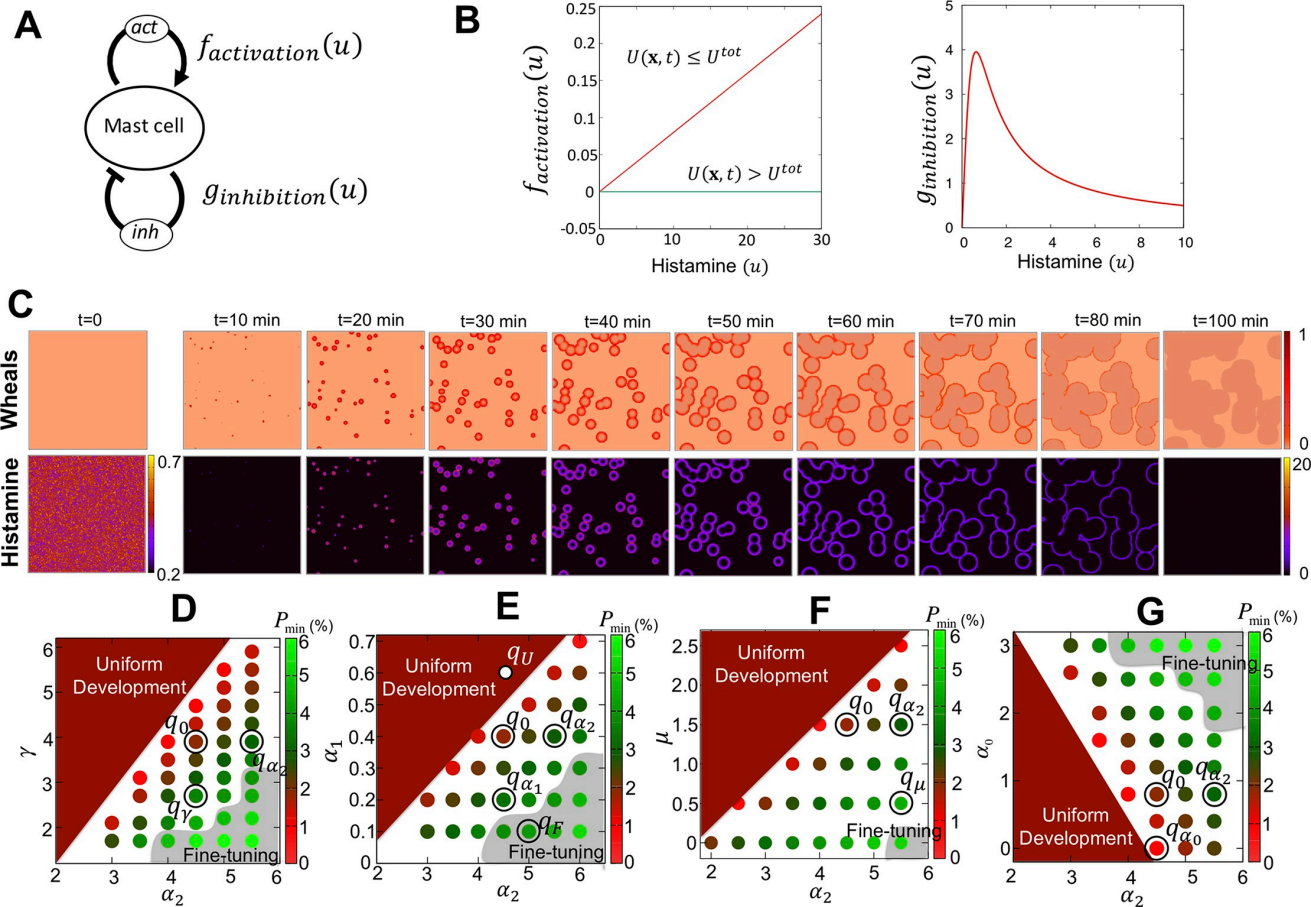
Model assumptions and simulation result. (A, B) Self-activation and self-inhibition loops of histamine via a mast cell, and the detailed functions of the loops. The example graphs are drawn for *γ* = 0.008, *α*_1_ = 0.4, *α*_2_ = 5.0 in model (1). (C) A representative simulation result of the model (1) for wheals and histamine. The parameter is assigned to Table 1 (Set I). The initial condition is shown in the figure at *t* = 0 for histamine. (D-G) Parameter spaces for wheal development with respect to *P*_*min*_ are plotted by the color bar. Uniform development is the region where wheals are uniformly developed in the area corresponding to no pattern state. Fine-tuning is the region where wheals developed as very fine dots with diameters less than 3 mm and were widely spread over the area corresponding to eruptions shown in Fig 3. *q*_0_, $q_{\alpha_{1}},\;q_{\alpha_{2}},\;q_{\alpha_{0}}$, *q*_*μ*_, *q*_*γ*_, *q*_*F*_, *q*_*U*_ are labels of parameter values chosen in the simulation results in (Fig 3A–3F).

To find a possible underlying mechanism that explains the dynamics of the multifarious eruptions in urticaria, we developed a simple conceptional mathematical model. A certain number of mast cells and blood vessels are sufficiently and uniformly distributed in the skin, as wheals observed in urticaria are not less than a few millimeters in diameter, where tens of mast cells reside [11]. Thus, we assume that mast cells are distributed spatially homogeneously in a simulation space $[0,L]\times [0,L]\subset R^{2}$. Next, we formulate the following reaction-diffusion equation by taking the concentration of histamine that represents mediators released from mast cells in the dermis as *u*(**x**,*t*) ($x=(x,y)\in R^{2}$) such that

$$\frac{\partial u}{\partial t}=D_{u}\nabla^{2}u+f_{activation}(u)-g_{inhibition}(u)+\mu -\alpha_{0}u$$ (1)

where *D*_*u*_ is the diffusion coefficient of histamine that has been quantitatively estimated from the experiments utilizing intradermal injection (See Methods in detail), *μ* is the basal release rate of histamine by mast cells, and *α*_0_ is basal decay rate of histamine. The key feature of our model is that the dynamics of mediators represented by histamine are regulated by two independent regulatory loops (Fig 2A). One is the self-activation loop of histamine (*f*_*activation*_(*u*)). Medically, wheals observed in urticaria is considered to develop by the cascaded positive feedback of histamine release from mast cells (Fig 1A) [4, 20], but the detailed network of molecular mechanism is still unknown. Thus, we assume the simplest feedback, such that histamine released from a mast cell induces further release of histamine via activating surrounding mast cells, which contain a limited amount of histamine [24]. Thus, we assume *f*_*activation*_(*u*) as

$$f_{activation}(u)=\gamma u\chi (U),$$

where *γ* is the rate of histamine release from a mast cell, and *χ*(*U*) is given by

$$\chi (U(x,t))=\left\{ \begin{array}{cc} 1 & if\;U(x,t)=\int_{0}^{t}\gamma u(x,t)dt\leq U^{tot}\\ 0 & else \end{array}\right..$$

*U*(**x**,*t*) indicates the total sum of released histamine from a mast cell during [0,*t*] and *U*_*tot*_ is the total amount of histamine contained initially in the mast cell (Fig 2B Left and see Methods for more details of model formulation). The other is the inhibition loop (*g*_*inhibition*_(*u*)) where we assume that histamine inhibits the activity of skin mast cells in a dose-dependent manner which results in the decrease of histamine concentration in dermis when it is less than a threshold, but this effect decreases once the amount of histamine becomes larger than the threshold. Thus, we assume *g*_*inhibition*_(*u*) in the simplest form as

$$g_{inhibition}(u)=\frac{\alpha_{2}u}{\alpha_{1}+u^{2}}$$

where *α*_1_ is the positive constant implying the histamine concentration where the inhibition effect is the highest, and *α*_2_ is the positive constant determining the highest level of inhibition effect (Fig 2B Right). For the inhibitory regulation, we assume that there exists a self-inhibition mechanism which is directly regulated in a histamine concentration-dependent manner via mast cells [23,25,26]. That is, the production of the inhibitory mediator is activated and the self-inhibitory effect increases in a concentration-dependent manner when the histamine concentration is less than a threshold, but is suppressed and the self-inhibitory effect decreases, once the amount of histamine becomes larger than the threshold. The specific inhibitory substance that directly acts on histamine dynamics via mast cells is still unknown, and the detailed form of inhibitory function above is fully a model assumption. However, certain substances derived from mast cells inhibit the release and/or effect of histamine synthesized by mast cells as referred in the Introduction section and will be discussed in more detail in the Discussion section [22, 23, 25].

Next, we set up an initial stimulus implying an external stimulus, such as allergens. Since we consider the urticaria occurred in a wide region of body by non-local stimulus, the mathematical setup for initial condition is given to a spatially perturbed function around the minimal homogeneous steady state ($u_{0}^{*}$) of histamine concentration *u*(**x**,*t*) such that

$$u(x,0)=u_{0}^{*}+r\frac{\phi (x)+s\psi (x)}{1+s}$$

where *ϕ*(**x**) is the perturbation function given to random values within [0, 1] and *ψ*(**x**) is the weight function defining a long-range spatial distribution of the stimulus. For *ψ*(**x**), we used the following equation:

$$\psi (x)=\frac{\cos\left(\frac{n\pi x}{L}\right)\cos\left(\frac{m\pi y}{L}\right)+1}{2}$$

where *n* and *m* are integers and *L*×*L* is the length of simulation space. *r* and *s* are positive parameters representing the (maximal) length of given perturbation (stimulus size) and weight strength, respectively. We also defined the strength of initial stimulus by *P*_*r*_ such that

$$P_{r}=\frac{r}{Maximal\;concentration\;of\;histamine}(\%),$$

and the minimal strength of initial stimulus at which urticaria develops by *P*_*min*_. Since urticaria is a phenomenon confined within the human body, we assumed zero flux boundary conditions.

Finally, we define the wheal state function. The averaged estimation of wheal radius development has shown that the size of the wheal converges to a constant value and the expansion speed becomes almost zero (Fig 1B and S1B Fig). This indicates that there is a threshold value of histamine concentration (denoted by *u*_*r*_) for wheal development. That is, when the histamine concentration is less than *u*_*r*_, the wheal expansion does not occur. Based on these features, we defined a function transferring the histamine concentration to wheal state under the following two assumptions: The first is that the wheal dynamics are simply reflected by the histamine dynamics, and we neglected the delay between the histamine and wheal dynamics. Thus, we assumed that the time scale of wheal dynamics in the skin is supposed to be similar to that of histamine dynamics in the dermis. The other assumption is that a wheal notably appears when the histamine concentration exceeds a threshold concentration [27]. Based on these assumptions, we defined the wheal state function that can directly reflect the dynamics of histamine as follows.

$$H(u)=\frac{1}{1+exp[-\beta (u-u_{r})]}$$

where *β* is a positive constant. This function allows two states of wheal states, namely, wheal present (1) /absent (0), and wheals appear when the histamine concentration is larger than the threshold, *u*_*r*_.

### Regeneration of CSU by self-activation and self-inhibition regulations

Using the mathematical model, we first found the dynamics of wheals that were similar to those observed in real patients in a clinical setting (Fig 2C and S1 Movie), where the annular pattern was observed, and the small ring patterns grew up and fused, resulting in an uneven and irregular pattern. We also found that there is a threshold value of initial stimulus that causes urticaria to develop (S1 Text and S3A Fig). We, thus, explored how a minimal size of stimulus, *P*_*min*_ at which urticaria develops, is related to the parameters in the model (1). We found that *P*_*min*_ varies depending on the parameters (Fig 2D–2G) and that wheals do not tend to emerge when the parameters of positive feedback (*γ*,*μ*) are low and the parameters of negative feedback (*α*_0_,*α*_2_) are high. By using numerical and linear stability analysis, we also found that wheals do not emerge when *γ*−*α*_0_<0. This condition includes the case of *γ* = 0, namely, activation absent case (S1 Text and S3B and S3C Fig), implying that the self-activation effect is indispensable for the wheal development of urticaria. Moreover, when the inhibition rate (*α*_2_) is low, wheals develop with very small *P*_*min*_ (the parameter region of uniform development). With no inhibition effect (namely, *α*_2_ = 0), our model becomes a linear equation and the histamine increases exponentially without spatial heterogeneity, once wheals emerged (*γ*−*α*_0_≥0) (S1 Text and S3E Fig). Taken together, we concluded that the activating regulation plays an important role in the emergence of wheal, while the inhibitory dynamics of histamine plays a key role in the spatial heterogeneity of wheals in urticaria.

### Multifarious eruptions of urticaria explained by model parameters and initial stimulus

**Fig 3:**
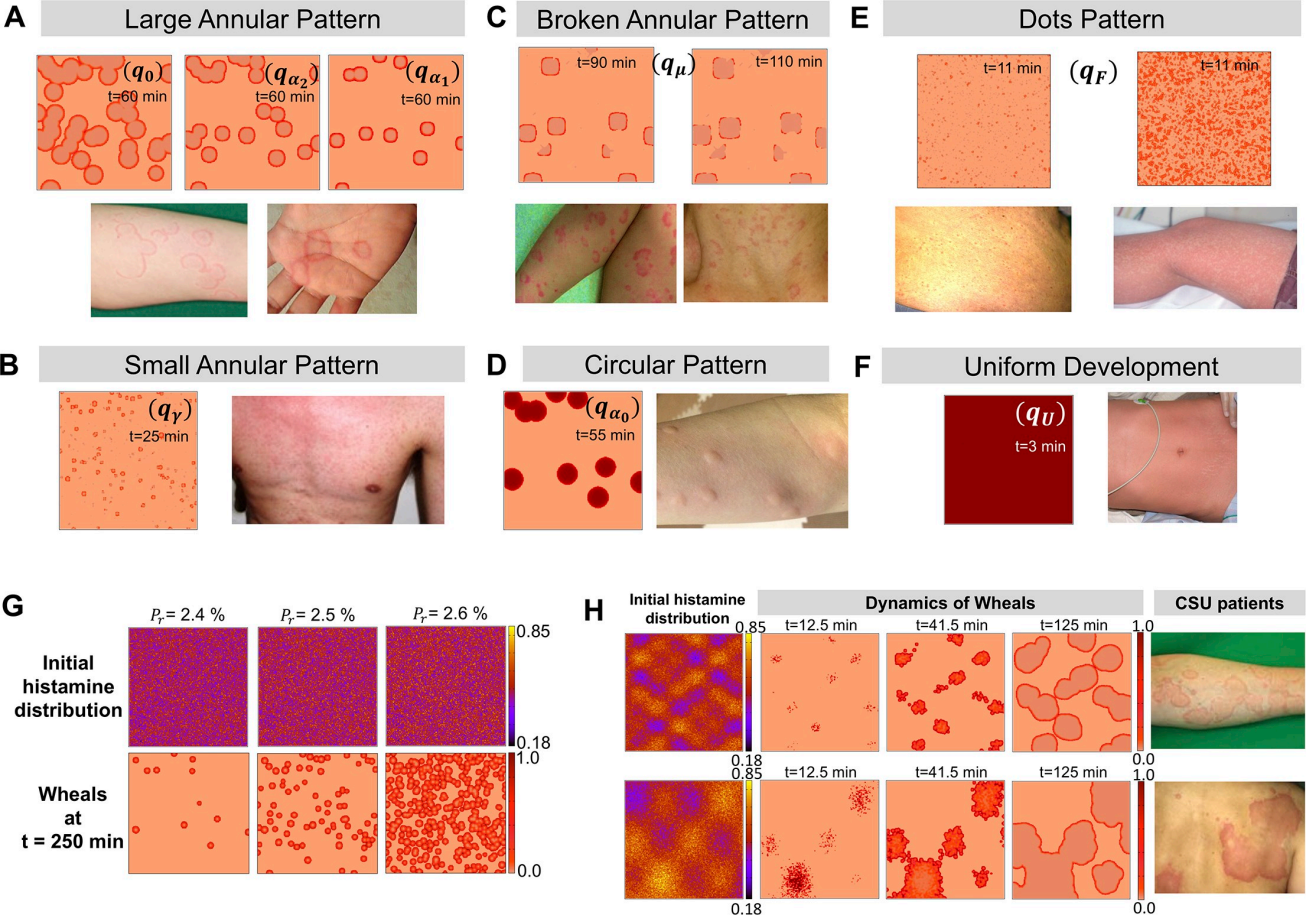
Multifarious eruptions of urticaria and simulation results. (A-F) Patterns of representative urticaria eruptions developed in patients (lower or right panels), and patterns simulated (upper or left panels) by parameters notated by (*q*_0_), $\left(q_{\alpha_{1}}\right)$, $\left(q_{\alpha_{2}}\right)$, $\left(q_{\alpha_{0}}\right)$, (*q*_*μ*_), (*q*_*γ*_), (*q*_*F*_), (*q*_*U*_) in (Fig 2D–2G) were assigned to similar eruptions developed in real patients with urticaria. The right-hand side figure in C was generated with a stronger initial stimulus than that in the left-hand side figure. The time of simulation result is notated in each figure. (G) Wheal patterns for the increase in the strength of the stimulus (*P*_*r*_). The density of wheals is notably increased. (H) Wheal patterns are shown with respect to two different weight functions of *ψ*(x) with the fixed *P*_*r*_. *α*_2_ = 5.0,u* = 0.181, and the other parameters chosen were listed in Table 1 (Set I) for (G) and (H).

Next, we investigated how the geometry of wheals may be affected by the parameter choice. The representative simulation results are shown in Fig 3A–3F and the chosen parameters were labelled as *q*_(parameter)_ in Fig 2D–2G. We found that the visible appearances of wheals could be classified into five types; large annular pattern, small annular pattern, broken annular pattern, circular pattern and dots pattern, corresponding to real eruptions observed in urticaria. When we decreased *α*_1_ or increased *α*_2_ from the representative parameter sets used in Fig 2C (*q*_0_ in Fig 2E), a large annular pattern was observed without change in the time course (Fig 3A). In contrast, a small annular pattern appeared when the histamine release rate (*γ*) was small (Fig 3B, S2 Movie and *q*_*γ*_ in Fig 2D). When the basal secretion rate (*μ*) decreased, the wheals were partially broken in their shape (Fig 3C, S3 Movie and *q*_μ_ in Fig 2F). When the basal decay rate (*α*_0_) was small, a circular pattern appeared (Fig 3D, S4 Movie and $q_{\alpha_{0}}$ in Fig 2G) and showed very slow extinction (S1 Text and S4 Fig). Moreover, there was a common region (gray regions shown in Fig 2D–2G) for all parameters where *α*_2_ was large and the *P*_*min*_ was large, and the wide-spread small dots pattern appeared (Fig 3E). When the strength of stimulus is large, the wheal pattern becomes very similar to that observed in cholinergic urticaria (Fig 3E right figure and S5 Movie). In contrast, with parameters in the region of uniform development (Fig 2D–2G), very fine patterning of histamine occurs and the wheals develop in an indistinguishable manner with high density (S1 Text and S3E Fig), resembling homogenous eruptions often found in patients with anaphylactic shock (Fig 3F).

Next, we investigated how the strength of the stimulus (*P*_*r*_) and spatial heterogeneity of the stimulus influenced the pattern of wheals. We found that the density of wheal patterning is sensitively dependent on the strength of the stimulus (Fig 3G). When the strength of the stimulus increased slightly, the density of wheal pattern notably increased. Moreover, the spatial heterogeneity of the stimulus can induce a large scale of flower patterning (Fig 3H and S6 Movie). The wheals developed first in the place where the stimulus was administered, and then small dots rapidly fused and formed large-scale islands.

### Estimation of expanding speed of wheals in urticaria vs intradermal injection experiment

**Fig 4:**
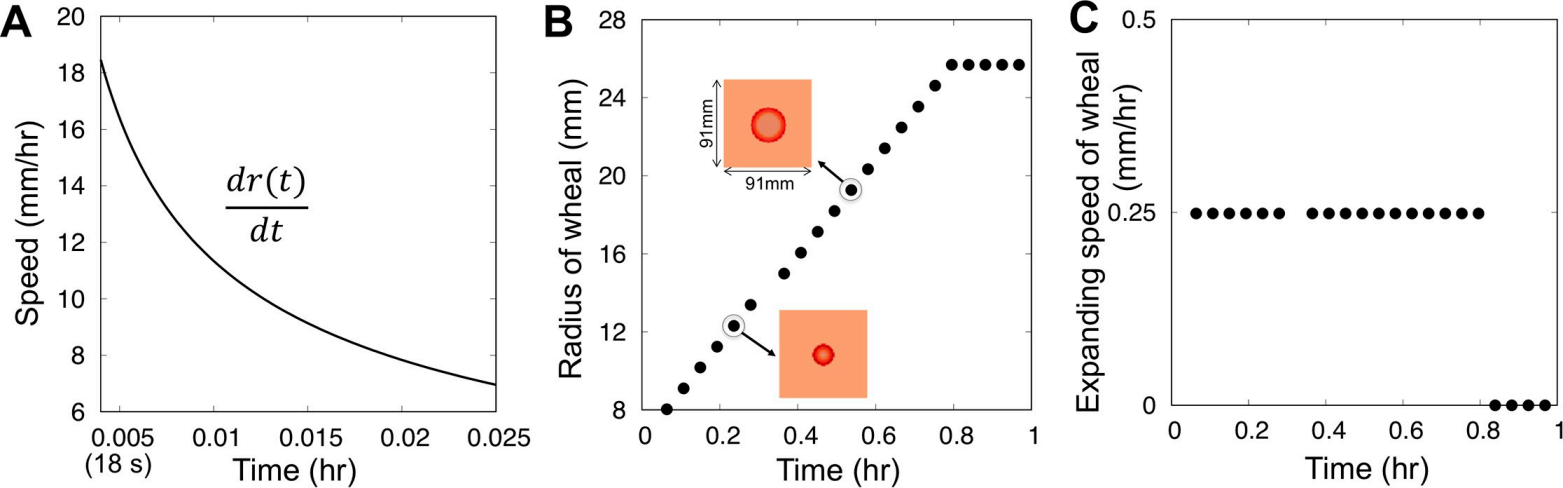
Comparison of the expanding speed of wheals developed by intradermal injection with that in urticaria using the mathematical model. (A) The expanding speed approximated by the Eq (4) for the injection of 10 *μg*/*ml* histamine. The maximal speed is determined in the initial stage. (B) Expanding radius of the wheal in the model (1). The parameters chosen were listed in Table 1 (Set I) (C) The expanding speed calculated from (B).

To explore how the activating and inhibitory mechanisms in our model can explain the difference in wheal expanding speeds observed in CSU and those in response to bolus intradermal injection of histamine, we compared the mathematically-estimated speed of wheal expansion by intradermal injection of histamine (Fig 1B and S1 Fig) and that in CSU by our mathematical model (Fig 4 and see Methods for detailed analysis) with those observed in real cases of urticaria and the intradermal injection experiments shown in Fig 1B and 1C. Our analysis showed that the expanding speed of wheal developed by intradermal injection decreased in monotone and the maximal speed of expansion is determined by the concentration of histamine given in the initial stage of injection (Fig 4A). In contrast, our model showed that the wheal expanding speed of CSU is constant (0.25 mm/hr) (Fig 4B and 4C) and much smaller than that by bolus injection of histamine. The estimated speeds are also very similar in scale to the results of the experiments and clinical observations shown in Fig 1C and S1 Fig.

When we consider the dynamics as a traveling wave, the expanding speed (*c*) can be analytically obtained such as

$$c\geq 2\sqrt{D(\gamma -\frac{\alpha_{2}(\alpha_{1}-u_{l}^{*2})}{{\left(\alpha_{1}+u_{l}^{*2}\right)}^{2}}-\alpha_{0})}$$

where $u_{l}^{*}$ is the unstable equilibrium in the model (See Supporting Information for the detailed analysis). It suggests that the expanding speed of wheal is increased as the rate of histamine release (*γ*) increases, but it is deceased as the basal decay rate of histamine (*α*_0_) or the highest level of inhibition effect (*α*_2_) increase. This result indicates that the inhibitory dynamics of histamine may account for the notable difference of wheal expanding speeds between those in the intradermal injection experiments and those observed in patients with urticaria.

## Discussion

Urticaria is a common skin disease but the underlying mechanism of the wheal formation has not been well understood. Our mathematical model suggests that not only a self-activation of histamine production via mast cells, but also its combination with self-inhibition in histamine dynamics plays a critical role in generating a wide-spread patterning of the wheal observed in urticaria, and that the balance of parameters may determine various geometries of the wheals. In contrast, the size and density of wheal patterns may be determined by the strength and randomness of the initial stimulus. In our model, the self-activation effect induced the increase of histamine and spread around cell with diffusion. If this positive feedback acts alone, the wheals emerge spatially uniformly by an external stimulus and all the skin will develop into the general rash condition as typically observed in anaphylaxis [28,29]. By contrast, the presence of self-inhibition may develop various spatial variations with the increase of histamine (Fig 2 and S3 Fig).

The presence of self-activation and self-inhibition mechanisms are supported by our recently published *in vitro* studies. Namely, histamine not only induces dilatation and hyperpermeability of the skin microvessels by itself at high concentrations, but it also triggers the extrinsic coagulation pathway in synergy with toll-like receptor agonists via the expression of tissue factor on vascular endothelial cells at low concentrations [25,26]. This reaction induces a small gap formation between endothelial cells in the presence of plasma coagulation factors [25,26]. This brings various histamine releasing factors to the blood, such as activated coagulation factors to activate protease activating receptor (PAR) on mast cells, autoantibodies against IgE or the high affinity IgE receptor (FcεRI) and autoantigens for IgE to the tissue. These factors should induce further release of histamine from mast cells in the vicinity, as well as neuropeptides released from free nerve endings in the skin by histamine [20, 30–35]. On the other hand, the expression of tissue factor on endothelial cells and histamine release from mast cells are consequentially suppressed by adenosine derived from ATP which is released from mast cells together with histamine [25,26]. Moreover, adenosine inhibits mast cell degranulation by itself [23].

We also found that a threshold of initial stimulus for the development of wheals, namely *P*_*min*_, varies depending on the parameters. Since *P*_*min*_ is defined as the ratio of histamine release rate from mast cells to the maximal concentration of histamine, histamine release over the threshold is necessary for the development of wheals in the initial phase. It is noteworthy that only a few percent release of the histamine released from mast cells may reach *P*_*min*_ and trigger wheal formation in this model. In a clinical setting, antihistamines compete with histamine and completely abolish the development of wheals for a large number/proportion of patients with urticaria, especially for those with CSU. This effectiveness is well explained by the reduction of histamine activity to a level lower than *P*_*min*_. In the case of the dot pattern wheals in which *P*_*min*_ is large, histamine concentration that evoked wheal formation must be high, and thus antihistamines may not compete with the activity of histamine to fall the levels below *P*_*min*_, resulting in no or only a small quantitative decrease in eruptions. Therefore, urticaria, such as cholinergic urticaria, that presents wheals of the dot pattern nature and presumably those of other types of inducible urticaria may be intractable with respect to treatment with antihistamines [36]. Thus, the characteristic of wheal patterning of individual patients could be a measure to classify the pathophysiology of urticaria, and the values of wheal parameters may be considered with respect to the choice of treatment for individual patients.

In this mathematical formula, we assumed histamine to be a mediator that is released from and acts on mast cells by itself. However, any substance that is released from mast cells together with histamine and that acts to both activate and inhibit may be plausibly included in the model assumption. In fact, histamine is released from mast cells in parallel to ATP, which is metabolized to adenosine, and consequentially inhibits mast cell activation both directly and indirectly as described above [25,26].

Our model is still conceptual at present and might not yet be directly applicable to real clinical management. Moreover, we did not take into account the synthesis of histamine which increases the amount of intracellular histamine. Nevertheless, the analysis of various clinical appearances of urticaria in accordance with this model and fine-tuning of the model should be a basis for understanding the pathogenesis of urticaria and aid decision-making for appropriate treatments. Moreover, the model may be extended to include skin structures using particle model [37] and the dynamics of immune system.

Urticaria is a common skin disease, and various factors are known to be associated with the onset and/or the aggravation of urticaria. Recent development of biologics, such as omalizumab, humanized monoclonal antibody against IgE, have largely improved its treatment [38,39]. However, many patients still do not benefit from or tolerate such therapies, and the molecular mechanism of wheal formation in urticaria is poorly understood [1,2,5]. It is largely due to a lack of proper experimental, small animal models for urticaria. Our mathematical model suggests a new possible mechanism of histamine to cause urticaria at a molecular level and may explain multifarious eruptions by two key dynamics; self-activating and self-inhibiting regulation of histamine. Moreover, our modeling framework suggests a new potential of a single reaction-diffusion equation as a new type of pattern-forming dynamics which creates diverse pattern transitions. The combination of a mathematical model with machine learning for pattern recognition may also help such procedures. Further studies of urticaria with specific parameters using our mathematical model may classify symptoms of urticaria in terms of characteristics of the underlying mechanisms of wheal formation, and be applicable to better precision medicine for individual patients.

We believe that our study could lead to a promising route to find molecules which may play a role as an inhibitory regulator in the pathogenesis of urticaria. It may also help in developing new treatments of skin diseases from a geometrical point of view through *in silico* experiments which has not been previously tried in medicine.

## Methods

### Time courses of wheals induced by the intradermal histamine injection

Eight healthy volunteers (M:F 7:1; mean age 36.8, range 28–59) were recruited in this experiment. No volunteers took anti-histamines. Wheals were induced on their forearm by the intradermal injection of 0.02 *ml* histamine at concentrations of 3, 10 and 30 *μg*/*ml*, and recorded on a digital camera at the indicated time points over 20 minutes (S1A Fig). The area of wheal (*A*_*w*_) was calculated from the number of pixels occupying the target wheal on the digital image with reference to the number of pixels in the standard area near the wheal. The radius of wheal (*R*_*w*_) was acquired by substituting *A*_*w*_ into the equation of *A*_*w*_ = *π*(*R*_*w*_)^2^. The regression analysis was applied to the *R*_*w*_ plotted in chronological order on a graph by the statistic software, Graphpad prism7 (GraphPad Software, Inc., CA, USA). Finally, the derivative of the regression curve of *R*_*w*_(*dR*_*w*_/*d*_*t*_) was defined as the radial expansion velocity of the wheal.

### Time courses of wheals in patients with CSU

Forty-nine wheals in 14 patients with chronic spontaneous urticaria (M:F 5:9; mean age 52.4, range 22–71) were analyzed under the approval by the ethics committee at Hiroshima University Hospital. Wheals spontaneously emerging on patients were recorded on a digital camera over multiple time points. The area of the urticarial wheal ($A_{w}^{u}$) was calculated from the number of pixels occupying the target wheal on the digital image with reference to the number of pixels in the standard area. Then, the radius of the wheal ($R_{w}^{u}$) was acquired by substituting $A_{w}^{u}$ into the equation of $A_{w}^{u}=\pi {\left(R_{w}^{u}\right)}^{2}$. The radial expansion velocity of wheal was calculated as the average during the observation time of two adjacent points.

### Model assumption for self-activation loop of histamine (*f*_*activation*_(*u*))

Let us denote the concentration of histamine contained in a mast cell at **x** and time *t* by *u*_*cell*_(**x**,*t*). Then, the concentration of histamine contained in a mast cell at **x** and time *t* can be written by

$$u_{cell}(x,t)=u_{cell}(x,t-\Delta t)-\Delta t[Released\;histamine\;amount\;from\;a\;mast\;cell\;per\;unit\;time].$$

We here assume that the released histamine amount from a mast cell per unit time is proportional to the concentration of extracellular histamine at **x** and time *t*. That is, we assume that the time interval between the time that a mast cell is stimulated and the time that histamine is released from a mast cell by the stimuli is sufficiently short and can be negligible. Then, the formula above can be written by

$$\frac{u_{cell}(x,t-\Delta t)-u_{cell}(x,t)}{\Delta t}=\gamma u(x,t)$$

where *γ* is a proportional constant. We here can define *γ* as the release rate. Therefore, with Δ*t*→0, the total released histamine amount from a mast cell during [0, t*] at each mast cell is given to

$$\int_{0}^{t^{*}}|\frac{u_{cell}(∙,t)}{dt}|dt=\int_{o}^{t^{*}}\gamma u(∙,t)dt.$$

Note that the left side term implies the total amount of released histamine from a mast cell during [0, t*]. Because the amount of histamine contained in a mast cell is limited, we obtain the following condition

$$\int_{o}^{t^{*}}\gamma u(∙,t)dt\leq U^{tot}.$$

The variation amount of extracellular histamine by the release from a mast cell at **x** is also given to

$$\frac{du(x,t)}{dt}=-\frac{u_{cell}(x,t)}{dt}=\gamma u(x,t).$$

### Quantitative analysis for estimation of diffusion coefficient of histamine

To estimate the diffusion coefficient of histamine in the dermis, we used the data from intradermal injection experiments of histamine (Fig 1B and 1C, S1 Fig and S1 Table). In the intradermal injection experiments, wheals do not spread around the whole body but remained localized, indicating that mast cells are not stimulated at single site so that the effects of self-activating and self-inhibiting loops in our model can be neglected. We also assume that the basal release and decay rates are in the equilibrium state and are maintained because the histamine injection is sufficiently localized. Thus, we assume that the disperse dynamics of histamine by intradermal injection is dominated by the diffusion equation. Because the formation of wheals by histamine injection in dermis is rapid, we assume that the dispersal of wheals occurs in a similar time scale with the dispersal of histamine in the dermis. Thus, we detected the diffusion coefficient of histamine by using the dynamics of the wheal. Let us assume that the dynamics of histamine in intradermal injection satisfies the diffusion equation as follows.

$$\frac{\partial u}{\partial t}=D_{u}\left(\frac{\partial^{2}u}{\partial x^{2}}+\frac{\partial^{2}u}{\partial y^{2}}\right).$$ (2)

With the initial condition *u*(*x*,0) = *u*_0_*δ*(*x*), the solution of the Eq (2) is given to

$$u(r,t)=\frac{u_{0}}{4D_{u}\pi t}\exp\left(-\frac{r^{2}}{4D_{u}t}\right)$$ (3)

where $r=\sqrt{x^{2}+y^{2}}$.

Let us define the radius of histamine wave at which the concentration of the histamine is *u*_*r*_ by r* (S2A Fig). Then we can see that r* has a critical value ($r_{max}^{*}$) at some time t* (S2B Fig), implying that the wheal in the intradermal injection experiment disperses, but stops in a limited size. That is,

$$u_{r}=\frac{u_{0}}{4D_{u}\pi t^{*}}\exp\left(-\frac{{r_{max}^{*}}^{2}}{4D_{u}t^{*}}\right)$$ (4)

is hold. Therefore, using the quantitative data and the Eq (4), we can estimate *D*_*u*_ if we can measure the values of *u*_*r*_, t* and $r_{max}^{*}$. However, detecting *u*_*r*_ is not easy in the intradermal experiments. We thus have estimated *D*_*u*_ without knowing the detailed value of *u*_*r*_ in the following method.

First, to find t* we have estimated the averaged graphs of *A*_*w*_,*R*_*w*_,*dR*_*w*_/*dt* of the intradermal injection experiment from calculating the averaged values of fitting functions for each subjective (S2C Fig), which is given by S1 Table. Using these data, we next have estimated t* and $r_{max}^{*}$ as the following steps.

(A) Finding the time t* at which the wheal expansion stops. t* is given to

$$t^{*}=\underset{t>0}{\min}\left\{t|\frac{dR_{w}}{dt}\leq \epsilon \right\}\;because\frac{d}{dt}\left(\frac{dR_{w}}{dt}\right)<0.$$

where *ϵ* is sufficiently small. We chose *ϵ* = 0.005(*mm/min*) which has been approximated by the smallest size of pixels in imaging analysis of the intradermal injection data.

(B) Calculate $r_{max}^{*}$ using $r_{max}^{*}=R_{w}(t^{*})$.

(C) Estimate *D*_*u*_ using the analytical solution (3) for each case: 3, 10, and 30 (*μg*/*ml*) as the following; let us denote the diffusion coefficients of 3, 10, and 30 (*μg*/*ml*) cases by *D*_1_,*D*_2_,*D*_3_. Because *u*_*r*_ should be same for the cases of 3, 10, and 30 (*μg*/*ml*), we have the following equations.

$$\frac{3(\mu g/ml)u_{nd}}{4D_{1}\pi t_{3(\mu g/ml)}^{*}}\exp\left(-\frac{r^{2}}{4D_{1}t_{3(\mu g/ml)}^{*}}\right)=\frac{10(\mu g/ml)u_{nd}}{4D_{1}\pi t_{10(\mu g/ml)}^{*}}\exp\left(-\frac{r^{2}}{4D_{1}t_{10(\mu g/ml)}^{*}}\right)$$

$$\frac{3(\mu g/ml)u_{nd}}{4D_{2}\pi t_{3(\mu g/ml)}^{*}}\exp\left(-\frac{r^{2}}{4D_{2}t_{3(\mu g/ml)}^{*}}\right)=\frac{30(\mu g/ml)u_{nd}}{4D_{2}\pi t_{30(\mu g/ml)}^{*}}\exp\left(-\frac{r^{2}}{4D_{2}t_{30(\mu g/ml)}^{*}}\right)$$

$$\frac{10(\mu g/ml)u_{nd}}{4D_{3}\pi t_{10(\mu g/ml)}^{*}}\exp\left(-\frac{r^{2}}{4D_{3}t_{10(\mu g/ml)}^{*}}\right)=\frac{30(\mu g/ml)u_{nd}}{4D_{3}\pi t_{30(\mu g/ml)}^{*}}\exp\left(-\frac{r^{2}}{4D_{3}t_{30(\mu g/ml)}^{*}}\right)$$

where *u*_*nd*_ is the scaling parameter for the initial concentration of histamine. By taking the mean of *D*_1_,*D*_2_, and *D*_3_ solved from the above equations, we estimated *D*_*u*_. The detailed values which we estimated by above steps are given in S2 Table.

S2D Fig shows how much the estimated diffusion coefficient of histamine fits the patient data. Using the Eq (4) and the initial wheal size (*r*_0_) in the intradermal experiment data, we obtained the evolution graph of the wheal radius as the following.

$$r_{max}^{*}(t)=2\sqrt{D_{u}t\;\log\left(\frac{u_{0}}{4D_{u}\pi u_{r}t}\right)}+r_{0},$$ (5)

in which the detailed value of *u*_*r*_ was calculated from the Eq (4) with data of S2 Table (*u*_*r*_ = 0.003748). In S2D Fig, we have approximated *u*_0_ = 0.3. In fact, from this approximation we can also obtain *u*_*nd*_ = 0.03 (*ml*/*μm*) because *u*_0_≈*u*_*nd*_×10 (*μg*/*ml*). Thus, we can also obtain $r_{max}^{*}(t)$ graphs for 3 (*μg*/*ml*) and 30 (*μg*/*ml***)** cases.

### Analysis of expanding speed of wheal vs intradermal injection experiment using mathematical models

From the Eq (5) and the detailed parameter values used in S2D Fig, we can directly calculate the expanding speed of the wheal radius ($dr_{max}^{*}/dt$) of the 10(*μg*/*ml*) intradermal injection experiment. This is likely to be explained by that the histamine dynamics of intradermal injection that can be assumed as a simple diffusion equation, where the expanding speed depends on the initial amount of histamine given in a local area (Eq (5) and Fig 4A).

In the mathematical model, we investigated the expanding speed of annular patterns (Fig 4B). We gave a *P*_*min*_ stimulation in the center of simulation space and calculated the expanding radius after the wheal has approached in a measurable size (Fig 4B). We found that the expanding speed is determined constantly until the total amount of histamine in a mast cell has been released out all (Fig 4C). Similarly, with the experimental result of Fig 1B, the wheal expanding speed in our model also showed much smaller speed than the maximal speed of intradermal injection case, and the value of speed was in a feasible range as shown in urticaria data (Fig 1C).

### Choice of model parameters

In order to visualize the global dynamics of wheals, we have chosen the spatial length scale as 27.4 cm×27.4 cm in the numerical simulations with the time scale of 250 seconds. We have quantitatively estimated the diffusion coefficient of histamine by using mathematical model and intradermal injection experiment data (See Methods). On the other hand, the details of kinetic parameters are completely unknown. Thus, we have investigated the model dynamics for the wide range of parameter values and qualitatively compared the model dynamics with real clinic data of urticaria. The representative range of parameters are given in Fig 2D–2G and the details of parameter values we used for specific figures in this paper are shown in Table 1.

**Table 1:** The representative parameter values used in simulations.

Parameters	Dimensionless value	Dimensional value	Set I
Spatial length (*L*×*L*)	1×1	27.4×27.4 (cm^2^)	27.4^2^ (cm^2^)
Time scale (*t*)	1.0	250 (sec)	250 (sec)
Diffusion rate (*D*_*u*_)	4.7×10^−6^	1.412×10^−5^(cm^2^/sec)	1.412×10^−5^(cm^2^/sec)
Histamine release rate (*γ*)	[0.0, 6.0]	[0.0, 0.012] (sec^-1^)	4.0 (0.008 sec^-1^)
Parameter affecting the gradient of inhibition rate (*α*_1_)	[0.0, 0.7]	[0.0, 0.028] (sec^-1^)	0.4 (0.0016 sec^-1^)
Parameter affecting the maximal inhibition rate (*α*_2_)	[0.0, 6.0]	[0.0, 0.024] (sec^-1^)	4.5 (0.018 sec^-1^)
Basal decay rate of histamine (*α*_0_)	[0.0, 3.0]	[0.0, 0.012] (sec^-1^)	0.7 (0.0028 sec^-1^)
Basal secretion rate of histamine (*μ*)	[0.0, 2.5]	[0.0, 0.1] (sec^-1^)	1.5 (0.06 sec^-1^)
Total amount of histamine (*U*^*tot*^)	[100, 600]	[5×10^4^,3×10^5^] (sec)	150(7.5×10^4^sec)

### Ethics statement

This study was performed under the approval by the ethics committee at Hiroshima University Hospital (The approval number, E-1008). Informed consents were obtained in the form of opt-out on the web-site.

## Supporting information

S1 Text Supplemental text. (DOCX)

S1 Fig Intradermal histamine injection experiments and wheal state function. (A) A representative time course of wheals induced by intradermal histamine injection, observed in a healthy volunteer (M.M.). Photographic images were arranged according to a time course and histamine concentrations. (B) The expansion of wheal radius (*R*_*w*_) was estimated from the area of wheal in (A) (See Methods). The solid lines are the averaged line for several numbers of subjects which have been obtained by regression analysis. The wheal radius was plotted by time and fitted into the curve of *Y* = *Y*_0_+(*P*−*Y*_0_)(1−*e*^−*αt*^). The maximum wheal radius increased in a dose-dependent manner of histamine concentration. The detailed fitting functions are shown in S1 Table. (C) The wheal state function. To represent that the complete recovery of skin takes time, we chose *β* to be smaller in case the histamine was in increasing state (red line) rather than the case that histamine concentration was in a decreasing state (green line), because the wheal disappears with the decrease of histamine concentration while complete recovery to the original skin state requires more time.
(TIFF)

S2 FigData of histamine injection experiments and diffusion estimation.(A) The solution of the diffusion equation *du*/*dt* = *D*∇^2^*u*, *u*(**x**,0) = *u*_0_*δ*(**x**) for *t* = *t*_1_,*t*_2_(*t*_1_<*t*_2_). r*(*t*) is defined by the radius at which $u(r,t)=u_{r}(r=\sqrt{x^{2}+y^{2}})$. (B) The graph of r*(***t***). There exists the maximal value $r_{max}^{*}$ of r*(*t*). t* is the time when $r^{*}(t)=r_{max}^{*}$. (C) The averaged values of *A*_*w*_ (the area of wheal *R*_*w*_), (the radius of wheal), *dR*_*w*_/*dt* (the speed of wheal expansion) for histamine injection experiment. (D) Comparison between the experiment data of CSU subjects (1 to 7) for 10 *μg*/*ml* and the radius of wheals (*R*_*w*_) obtained from the diffusion rate estimated from the experimental data (See Methods). The lines are given by the estimated equation $R_{w}=2\sqrt{Dt\;log(u_{0}/4D\pi u_{r}t)}+r_{0}$ where *D* = 0.08474977(*mm*^2^/*min*), *u*_*r*_ = 0.003748, *u*_0_ = 0.3, and *r*_0_ is given to the initial wheal size obtained in the experiment. The black bold line is *r*_0_ = 3.20875, and the thin blue lines are ***r***_**0**_ = 3.20875±0.42342. Each point indicates the experiment data of CSU patients.(TIFF)

S3 FigMathematical structure for urticaria emergence.(A) Wheal emergence/non-emergence depending on *P*_*r*_. The same parameters have been given for two simulations except for *P*_*r*_. *γ* = 4.0, *α*_1_ = 0.4,*α*_2_ = 5.0,*α*_0_ = 0.7,*μ* = 1.5 are chosen as nondimensional parameters. The initial condition was given with *s* = 0. (B) The example graphs of *du*/*dt* (i.e. the case of *D*_*u*_ = 0) for the size relation between histamine release rate (*γ*) and basal decay rate (*α*_0_). $u_{0}^{*}$ and $u_{l}^{*}$ are two positive equilibria. The white and black circles indicate a stable and unstable state in the given equilibrium, respectively. Arrows indicate the direction of histamine concentration around equilibria. (C) Parameter space for developing urticaria. In the black shaded region, uniform urticaria develops without a pattern. *α*_1_ = 0.4, *μ* = 1.5 are chosen in a nondimentional parameters. *γ*,*α*_2_,*α*_0_ are plotted with nondimensional scale. (d1) is (2.0, 3.0), (d2) is (5.0, 1.0), and (d3) is (5.0, 2.92). (D) The graphs of *du*/*dt* for each case, (d1), (d2) and (d3). (E) Time course of wheals and histamine distributions simulated by the parameter set of (d1) in C.(TIFF)

S4 FigWheal differences and temporal dynamics for circular wheal pattern.(A) The reaction terms of the equation (1.3) for annular patterns (red line) and circular patterns (green line). Shaded circle implies a stable equilibrium and blanked circle implies an unstable equilibrium. (B) The reaction term of the equation (1.3) for circular patterns with respect to the value of *α*_0_. *u*_−_ and *u*_+_ are stable equilibria. (C) Temporal dynamics for the case of $q_{\alpha_{0}}$ in Fig 3E.(TIFF)

S1 Table Fitting functions of *A*_*w*_,*R*_*w*_,*dR*_*w*_/*dt*. (DOCX)

S2 Table The detailed estimated values for histamine diffusion coefficient. (DOCX)

S1 Movie The representative simulation result of the model (1) for wheals. The case of large annular pattern.
(MOV)

S2 Movie Small annular pattern. (MOV)

S3 Movie Broken annular pattern. (MOV)

S4 Movie Circular pattern. (MOV)

S5 Movie Dots pattern. (MOV)

S6 Movie Large scale islands pattern. (MOV)

## References

[1] Lee SJ, Ha E.K., Jee HM, Lee KS, Lee SW, Kim MA, Kim DH, Jung YH, Sheen YH, Sung MS, Han MY, Prevalence and Risk Factors of Urticaria With a Focus on Chronic Urticaria in Children. Allergy Asthma Immunol Res. 2017; 9: 212–219. 10.4168/aair.2017.9.3.212 28293927 PMC5352572

[2] Maxim E, Aksut C, Tsoi D, Dellavalle R. Global burden of urticaria: Insights from the 2016 Global Burden of Disease Study. J. Am. Acad. Dermatol. 2018; 79: 567–569. 10.1016/j.jaad.2018.02.026 29438758 PMC6119764

[3] Maurer M, Weller K, Bindslev-Jensen C, Gimenez-Arnau A, Bousquet PJ, Bousquet J, Canonica GW, Church MK, Godse KV, Grattan CE, Greaves MW, Hide M, Kalogeromitros D, Kaplan AP, Saini SS, Zhu XJ, Zuberbier T. Unmet clinical needs in chronic spontaneous urticaria. A GA2LEN task force report. Allergy. 2011; 66(3): 317–30. 10.1111/j.1398-9995.2010.02496.x 21083565

[4] Church MK, Kolkhir P, Metz M, Maurer M, The role and relevance of mast cells in urticaria, Immunological Reviews 2018; 282: 232–247. 10.1111/imr.12632 29431202

[5] Itakura A, Tani Y, Kaneko N, Hide M, Impact of chronic urticaria on quality of life and work in Japan: Results of a real-world study. Journal of Dermatology. 2018; 45: 963–970. 10.1111/1346-8138.14502 29897137 PMC6099381

[6] Jain S. Pathogenesis of chronic urticaria: an overview. Dermatol Res Pract. 2014; 674709. 10.1155/2014/674709 PMC4120476 25120565

[7] Jaffar Z. H. and Pearce E. L., Histamine secretion from mast cells stimulated with ATP. Agent and Actions.1990; 30: 64–66. 10.1007/BF01968999 1695463

[8] Kurashima Y. and Kiyono H., New era for mucosal mast cells: their roles in inflammation, allergic immune responses and adjuvant development. Experimental & Molecular Medicine. 2014; 46: e83. 24626169 10.1038/emm.2014.7 PMC3972796

[9] Matsuo Y, Yanase Y, Irifuku R, Takahagi S, Mihara S, Ishii K, Kawaguchi T, Tanaka A, Iwamoto K, Watanuki H, Furuta K, Tanaka S, Inoue A, Aoki J, Hide M. Neuromedin-U directly induces degranulation of skin mast cells, presumably via MRGPRX2, Allergy. 2018;73(11): 2256–2260. 10.1111/all.13555 29987892

[10] Redegeld FA, Yu Y, Kumari S, Charles N, Blank U. Non-IgE mediated mast cell activation. Immunol Rev. 2018; 282(1): 87–113. 10.1111/imr.12629 29431205

[11] Bazilai A, Sagi L, Baum S, Trau H, Schvimer M, Barshack I, Solomon M, The Histopathology of Urticaria Revisited-Clinical Pathological Study. Am J Dermatopathology. 2017; 39: 753–759. 10.1097/DAD.0000000000000786 28858880

[12] Kaplan AP, Horakova Z, Katz SI. Assessment of tissue fluid histamine levels in patients with urticaria. J Allergy Clin Immunol 1978;61:350–4. 10.1016/0091-6749(78)90113-6 659726

[13] Guida B, De Martino CD, De Martino SD, Tritto G, Patella V, Trio R, et al Histamine plasma levels and elimination diet in chronic idiopathic urticaria. Eur J Clin Nutr. 2000;54(2):155–8. 10.1038/sj.ejcn.1600911 10694787

[14] Church MK, Kolkhir P, Metz M, Maurer M. The role and relevance of mast cells in urticaria. Immunol Rev. 2018;282(1):232–47. 10.1111/imr.12632 29431202

[15] Grattan C, Powell S, Humphreys F, British Association of Dermatologists. Management and diagnostic guidelines for urticaria and angiooedema. Br J Dermatol. 2001; 144: 708–714. 10.1046/j.1365-2133.2001.04175.x 11298527

[16] Greaves MW. Pathology and classification of urticaria. Immunol Allergy Clin North Am. 2014;34(1):1–9. 10.1016/j.iac.2013.07.009 24262685

[17] Petersen L. J., Church M. K. and Stahl Skov P., Histamine is released in the wheal but not the flare following challenge of human skin *in vivo*: a microdialysis study. Chemical and Experimental Allergy. 1997; 27: 284–295. 10.1046/j.1365-2222.1997.d01-502.x 9088655

[18] Hide M, Takahagi S. Urticaria and Angioedema in Fitzpatrick's dermatology. 9th ed. p 684–709, McGraw-Hill Education; 2019.

[19] Hide M, Suzuki T, Tanaka A, Aoki H. Efficacy of increased dose of rupatadine up to 20 mg on itching in Japanese patients due to chronic spontaneous urticaria, dermatitis, or pruritus: A post hoc analysis of phase III clinical trial. Journal of Cutaneous Immunology and Allergy. 2019;0(0). 10.1002/cia2.12072

[20] Rosa AC, Fantozzi R. The role of histamine in neurogenic inflammation. Br J Pharmacol. 2013;170(1):38–45. 10.1111/bph.12266 23734637 PMC3764847

[21] Murray JD, Mathematical Biology II: Spatial Models and Biomedical Applications, Springer, Berlin; 2003.

[22] Matsuo Y, Yanase Y, Irifuku R, Ishii K, Kawaguchi T, Takahagi S, Hide I, Hide M. The role of adenosine for IgE receptor-dependent degranulation of human peripheral basophils and skin mast cells. Allergol Int. 2018; 67(4):524–526. 10.1016/j.alit.2018.03.007 29703695

[23] Suender CA, Leist M, Abrink M, et al Mast cells are critical for the limitation of thrombin-induced skin inflammation. Exp Dermatol. 2018;27(1):50–57. 10.1111/exd.13407 28787094

[24] Hattori Y, Seifert R, Histamine and histamine receptors in health and disease (Part IV: Church MK, Allergy, histamine and antihistamines), Springer, Cham; 2017.

[25] Yanase Y, Morioke S, Iwamoto K, Takahagi S, Uchida K, Kawaguchi T, Ishii K, Hide I, Hide M. Histamine and TLR ligands synergistically induce endothelial-cell gap-formation by the extrinsic coagulating pathway. J Allergy Clin Immunol. 2018; 141(3): 1115–1118. 10.1016/j.jaci.2017.07.026 28859973

[26] Yanase Y., Takahagi S. and Hide M., Chronic spontaneous urticaria and the extrinsic coagulation system. Allergol Int. 2018; 67:191–194. 10.1016/j.alit.2017.09.003 28993062

[27] Zuberbier T., Greaves M. W., Juhlin L., Kobza-Black A., Maurer D., Stingl G., Henz B. M., Definition, classification, and routine diagnosis of urticaria: a consensus report. J Invest Dermatol Symposium Proceedings. 2001; 6:123–127. 10.1046/j.0022-202x.2001.00022.x 11764296

[28] Morimoto K, Tanaka T, Sugita Y, Hide M. Food-dependent exercise-induced anaphylaxis due to ingestion of orange. Acta Derm Venereol. 2004;84(2):152–153. 10.1080/00015550310006860 15206698

[29] Hirata JI, Ohya M, Kumon K. Diagnosis and long-term management of hydrolyzed wheat protein wheat-dependent exercise-induced anaphylaxis. Acute Med Surg. 2015;2(4):260–262. 10.1002/ams2.114 29123735 PMC5649279

[30] Cugno M, Tedeschi A, Frossi B, Bossi F, Marzano AV, Asero R. Detection of low-molecular-weight mast cell-activating factors in serum from patients with chronic spontaneous urticaria. J. Investig Allergol Clin Immunol. 2016; 26(5): 310–313. 10.18176/jiaci.0051 27763857

[31] Hatada Y, Kashiwakura J, Hayama K, Fujisawa D, Sasaki-Sakamoto T, Terui T, Ra C, Okayama Y. Significantly high levels of anti-dsDNA immunoglobulin E in sera and the ability of dsDNA to induce the degranulation of basophils from chronic urticaria patients. Int Arch Allergy Immunol. 2013;161 Suppl 2:154–8. 23711867 10.1159/000350388

[32] He SH, Xie H, Fu YL. Activation of human tonsil and skin mast cells by agonists of proteinase activated receptor-2. *Acta Pharmacol Sin*. 2005; 26(5): 568–574. 10.1111/j.1745-7254.2005.00079.x 15842775 PMC7091817

[33] Hide M, Francis DM, Grattan CE, Hakimi J, Kochan JP, Greaves MW. Autoantibodies against the high-affinity IgE receptor as a cause of histamine release in chronic urticaria. N Engl J Med. 1993; 328(22): 1599–1604. 10.1056/NEJM199306033282204 7683772

[34] Maurer M, Rosén K, Hsieh HJ, Saini S, Grattan C, Gimenéz-Arnau A, Agarwal S, Doyle R, Canvin J, Kaplan A, Casale T. Omalizumab for the treatment of chronic idiopathic or spontaneous urticaria. N Engl J Med. 2013; 368(10): 924–935. 10.1056/NEJMoa1215372 23432142

[35] Schmetzer O, Lakin E, Topal FA, Preusse P, Freier D, Church MK, Maurer M. IL-24 is a common and specific autoantigen of IgE in patients with chronic spontaneous urticaria. J Allergy Clin Immunol. In press. 10.1016/j.jaci.2017.10.035 29208545

[36] Kocatürk E, Can PK, Akbas PE, Copur M, Degirmentepe EN, Kızıltac K, Singer R. Management of chronic inducible urticaria according to the guidelines: A prospective controlled study. J Dermatol Sci. 2017 7;87(1):60–69. 10.1016/j.jdermsci.2017.02.283 28314658

[37] Kobayashi Y., Sawabu Y., Kitahata H., Denda M., Nagayama M., Mathematical model for calcium-assisted epidermal homeostasis. Journal of Theoretical Biology. 2016; 397: 52–60. 10.1016/j.jtbi.2016.02.032 26953648

[38] Min TK, Saini SS. Emerging Therapies in Chronic Spontaneous Urticaria. Allergy Asthma Immunol Res. 2019;11(4):470–81. 10.4168/aair.2019.11.4.470 31172716 PMC6557779

[39] Eyerich S, Metz M, Bossios A, Eyerich K. New biological treatments for asthma and skin allergies. Allergy. 2019 10.1111/all.14027 31444793

