# Supplementary material for: A single reaction-diffusion equation for the multifarious eruptions of urticaria
Source: PLoS Comput Biol. 2020 Jan 15;16(1):e1007590. doi: 10.1371/journal.pcbi.1007590 (PMC6961880; doi:10.1371/journal.pcbi.1007590)
Supplement: S1 Text — (DOCX) [file pcbi.1007590.s001.docx]

**S1 Text**

A Single Reaction-Diffusion Equation for the Multifarious Eruptions of Urticaria

Sungrim Seirin-Lee, Yuhki Yanase, Shunsuke Takahagi, Michihiro Hide

1. **Supplemental Text**
2. **Mathematical analysis of conditions for urticaria development**

We investigated how the magnitude of initial stimulus on a mast cell plays a role in development of urticaria (S3 Fig A). We found that histamine does not increase and stays in the initial stable equilibrium when $P_{r}$ is small as shown in S3 Fig A, and that there exists a threshold size of $P_{r}$ for the pattern emerging. In order to find the reason for that the development of the urticaria pattern is dependent on the size of $P_{r}$ we analyzed the equilibria and linear stability for the model

$$\frac{\partial u}{\partial t}=D_{u}\nabla^{2}u+f_{activation}\left( u \right)-g_{inhibition}\left( u \right)+\mu-\alpha_{0}u. (1.1)$$

S3 Fig B shows that there exist two cases: (i) Two positive equilibria exist when the release rate of histamine is equal to or larger than the basal decay rate of histamine ($\gamma\geq\alpha_{0}$); the smaller one is stable and larger one is unstable. (ii) There exists a unique positive equilibrium when the release rate of histamine is smaller than the basal decay rate of histamine ($\gamma<\alpha_{0}$); it is stable. For the case of (ii), we can easily prove it by reformulating the reaction term of (1.1) to polynomial form such that

$\left( \gamma-\alpha_{0} \right)u^{3}+\mu u^{2}+\left( \gamma\alpha_{1}-\alpha_{2}-\alpha_{0}\alpha_{1} \right)u+\alpha_{1}\mu=0$.

If $\gamma<\alpha_{0}$, there exist a single positive equilibrium and it is stable because $\alpha_{1}\mu>0$. In the case of (i), the release of histamine is not likely to increase if the initial state of histamine concentration is less than $u_{\mathcal{l}}^{*}$. This means that a strong stimulus on mast cells to release histamine more than $u_{\mathcal{l}}^{*}$ is required in order to develop urticaria. In the case of (ii), the histamine release does not increase from the initial state. Thus, $\gamma\geq\alpha_{0}$ is the basal condition for developing a pattern of urticaria, indicating that the self-activating system of histamine by mast cells is a core mechanism for emerging urticaria.

Next, we explored how the parameters are related to the condition for the development of the urticaria and $P_{r}$. As an example, we focused on $\gamma$, $\alpha_{0}$, and maximal inhibition rate of histamine, $\alpha_{2}$. We plotted the minimal value of $P_{r}$ (denoted by $P_{min}$) at which the wheal emerges, with changing values of $\kappa(=\gamma-\alpha_{0})$ and $\alpha_{2}$. As shown in S3 Figs C and D (d2, d3), $P_{min}$ increases as $\kappa$ decreases (i.e. either $\gamma$ is decreased or $\alpha_{0}$ is increased), indicating that the urticaria can easily develop by a small stimulus in the case of either large release rate or small basal decay rate. In contrast, we see that $P_{min}$ decreases as $\alpha_{2}$ decreases (S3 Figs C and D((d1), (d2))), implying that the urticaria can easily develop by a small stimulus as the inhibition rate of histamine is small.

Finally, we find that if $\kappa>\alpha_{2}$, there is no spatial pattern formation of histamine (S3 Figs C and E). It is also obvious that if we neglect the inhibition term, namely, $\alpha_{2}=0$, the model system (1.1) becomes a linear system such that

$$\frac{\partial u}{\partial t}=D_{u}\nabla^{2}u+\left( \gamma-\alpha_{0} \right)u+\mu,$$

and its solution will be divergent exponentially when $\gamma-\alpha_{0}>0$ and cannot create complex patterns [21]. This indicates that the inhibition effect plays an important role in creating a spatial heterogeneity of histamine distribution and the inhibition rate must not be small for the wheals patterning.

1. **Mathematical analysis of expanding speed of the wheal**

The expanding dynamics of wheals show that the hoop of wheal moves at constant speed as shown in Fig 4B. Thus, let us assume that the spreading dynamics is approximated by a traveling wave. For the analysis of the expanding speed, we first change the model equation with a polar coordinate, $x=r\sin\theta$, $y=r\cos\theta$ where $r=x^{2}+y^{2}$. Then, our main model with a positive release rate of histamine becomes

$$\frac{\partial u}{\partial t}=D_{u}\frac{\partial^{2}u}{\partial r^{2}}+\gamma u-\frac{\alpha_{2}u}{\alpha_{1}+u^{2}}+\mu-\alpha_{0}u, (1.2)$$

where we assumed that $r$ is sufficiently large. Let us $U\left( z \right)=U(r-ct)$ be a traveling wave solution satisfying $U\left( -\infty\right)=u_{1}^{*}$ and $U\left( +\infty\right)=u_{\mathcal{l}}^{*}$ where $u_{1}^{*}$ and $u_{\mathcal{l}}^{*}$ are the maximal stable equilibrium and an unstable equilibrium of (1.2), respectively. Then, we obtain

$$-c\frac{\partial U}{\partial z}=D_{u}\frac{d^{2}U}{dz^{2}}+\gamma U-\frac{\alpha_{2}U}{\alpha_{1}+U^{2}}+\mu-\alpha_{0}U,$$

which can be rewritten to

$$\frac{\partial U}{\partial z}=V,$$

$$\frac{\partial V}{\partial z}=-\frac{1}{D_{u}}\left( \gamma U-\frac{\alpha_{2}U}{\alpha_{1}+U^{2}}+\mu-\alpha_{0}U \right)-\frac{c}{D_{u}}V$$

with $V\left( \pm\infty\right)=0.$

Now let us suppose that histamine increases in monotone when the initial stimulus is sufficient. That implies that the edge of the wheal wave should not be oscillated. To find a mathematical condition satisfying this assumption, we carry out linear stability analysis at $\left( U, V \right)=\left( u_{\mathcal{l}}^{*}, 0 \right).$ The linearized system is given to

$$\left( \begin{matrix} U' \\ V' \end{matrix} \right)=\left( \begin{matrix} 0 & 1 \\ -\frac{A}{D_{u}} & -\frac{c}{D_{u}} \end{matrix} \right)\binom{U}{V}$$

where

$$A=\gamma-\frac{\alpha_{2}\left( \alpha_{1}-{u_{\mathcal{l}}^{*}}^{2} \right)}{\left( \alpha_{1}+{u_{\mathcal{l}}^{*}}^{2} \right)^{2}}-\alpha_{0}.$$

Then, the eigenvalue equation is given to

$$\lambda^{2}+\frac{c}{D_{u}}\lambda+\frac{A}{D_{u}}=0.$$

In order not to have an oscillating dynamics, the eigenvalues should not have an imaginary value and we have

$$\left( \frac{c}{D_{u}} \right)^{2}-4\frac{A}{D_{u}}\geq0.$$

Therefore, we obtain

$$c\geq2\sqrt{D\left( \gamma-\frac{\alpha_{2}\left( \alpha_{1}-u_{l}^{*2} \right)}{\left( \alpha_{1}+u_{l}^{*2} \right)^{2}}-\alpha_{0} \right)} .$$

1. **Mathematical analysis for types of wheals and temporal dynamics of urticaria**

Fig. 3 shows several types of wheal appearance. For the analysis of annular and circular ones, we consider the model when $\gamma=0$ as given to

$$\frac{\partial u}{\partial t}=D_{u}\frac{\partial^{2}u}{\partial r^{2}}-\frac{\alpha_{2}u}{\alpha_{1}+u^{2}}+\mu-\alpha_{0}u, (1.3)$$

In fact, the differences between annular and circular patterns are based on the stability of equilibria of the equation (1.3) as shown in S4 Fig A. In the case of annular patterns (red line in S4 Fig A), there exists one stable equilibrium of low concentrations of histamine, indicating that histamine quickly decreases after the total histamine content of a mast cell was released. In contrast, in the case of circular patterns (green line in S4 Fig A), there exists two stable equilibria, so that the histamine keeps a high concentration even though the total histamine content of a mast cell has been released out.

S4 Fig C shows that the extinction of the wheals becomes very slow when the basal decay rate is small. This can be understood by the propagation speed of a bi-stable reaction-diffusion equation [40]. Let us assume that the interface of the circular wheals is approximated to a propagation solution in an infinite domain, and the profile asymptotes to two bi-stable equilibria, $u_{-}$ and $u_{+}$. Then the speed of the front is determined by

$$c=\frac{\int_{u_{-}}^{u_{+}} f(u)du}{\int_{-\infty}^{+\infty} \left( \frac{du}{dr} \right)^{2}dr} .$$

Therefore, the value of $|\int_{u_{-}}^{u_{+}} f(u)du|$ determines the speed and we can see that this value becomes smaller as $\alpha_{0}$ becomes smaller in S4 Fig B.

**References**

1. Keener J, Sneyd J. Mathematical Physiology I: Cellular Physiology. Springer, New York; 2009.

S1 Table. Fitting functions of $\boldsymbol{A}_{\boldsymbol{w}}\boldsymbol{,}\boldsymbol{R}_{\boldsymbol{w}}\boldsymbol{,}\boldsymbol{dR}_{\boldsymbol{w}}\boldsymbol{/dt}$.

S2 Table. The detailed estimated values for histamine diffusion coefficient.
