# Supplementary figures and images for: A single reaction-diffusion equation for the multifarious eruptions of urticaria
Source: PLoS Comput Biol. 2020 Jan 15;16(1):e1007590. doi: 10.1371/journal.pcbi.1007590 (PMC6961880; doi:10.1371/journal.pcbi.1007590)

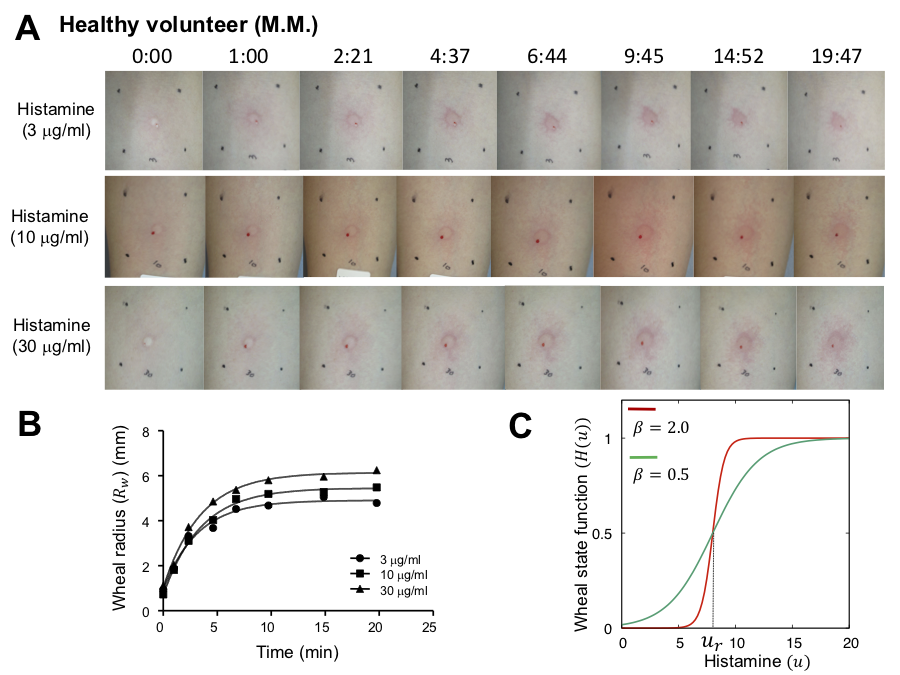

Supplement: S1 Fig — (A) A representative time course of wheals induced by intradermal histamine injection, observed in a healthy volunteer (M.M.). Photographic images were arranged according to a time course and histamine concentrations. (B) The expansion of wheal radius (Rw) was estimated from the area of wheal in (A) (See Methods). The solid lines are the averaged line for several numbers of subjects which have been obtained by regression analysis. The wheal radius was plotted by time and fitted into the curve of Y = Y0+(P−Y0)(1−e−αt). The maximum wheal radius increased in a dose-dependent manner of histamine concentration. The detailed fitting functions are shown in S1 Table. (C) The wheal state function. To represent that the complete recovery of skin takes time, we chose β to be smaller in case the histamine was in increasing state (red line) rather than the case that histamine concentration was in a decreasing state (green line), because the wheal disappears with the decrease of histamine concentration while complete recovery to the original skin state requires more time. (TIFF) [file pcbi.1007590.s002.tiff]

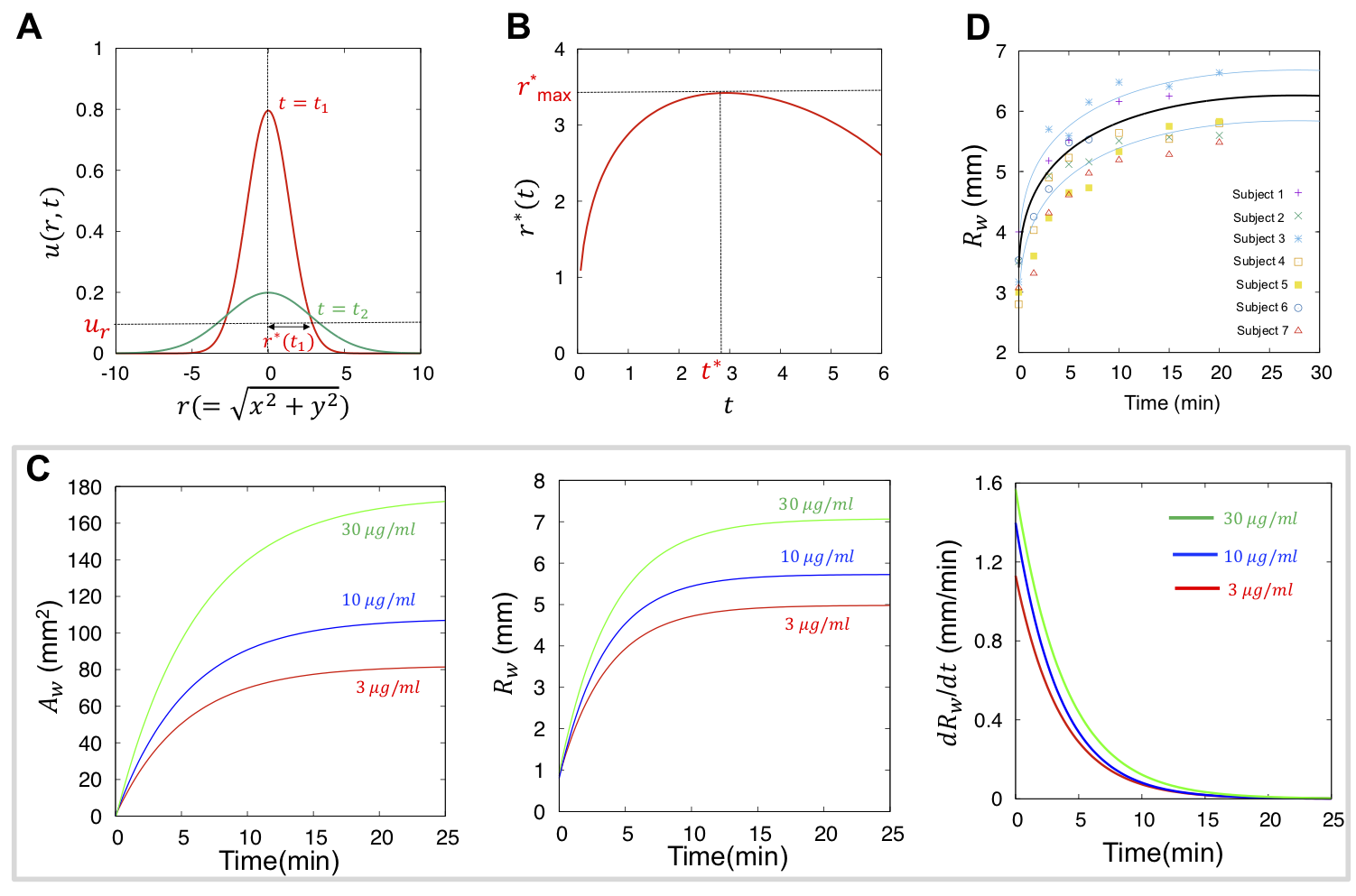

Supplement: S2 Fig — (A) The solution of the diffusion equation du/dt = D∇2u, u(x,0) = u0δ(x) for t = t1,t2(t1<t2). r*(t) is defined by the radius at which u(r,t)=ur(r=x2+y2). (B) The graph of r*(t). There exists the maximal value rmax* of r*(t). t* is the time when r*(t)=rmax*. (C) The averaged values of Aw (the area of wheal Rw), (the radius of wheal), dRw/dt (the speed of wheal expansion) for histamine injection experiment. (D) Comparison between the experiment data of CSU subjects (1 to 7) for 10 μg/ml and the radius of wheals (Rw) obtained from the diffusion rate estimated from the experimental data (See Methods). The lines are given by the estimated equation Rw=2Dtlog(u0/4Dπurt)+r0 where D = 0.08474977(mm2/min), ur = 0.003748, u0 = 0.3, and r0 is given to the initial wheal size obtained in the experiment. The black bold line is r0 = 3.20875, and the thin blue lines are r0 = 3.20875±0.42342. Each point indicates the experiment data of CSU patients. (TIFF) [file pcbi.1007590.s003.tiff]

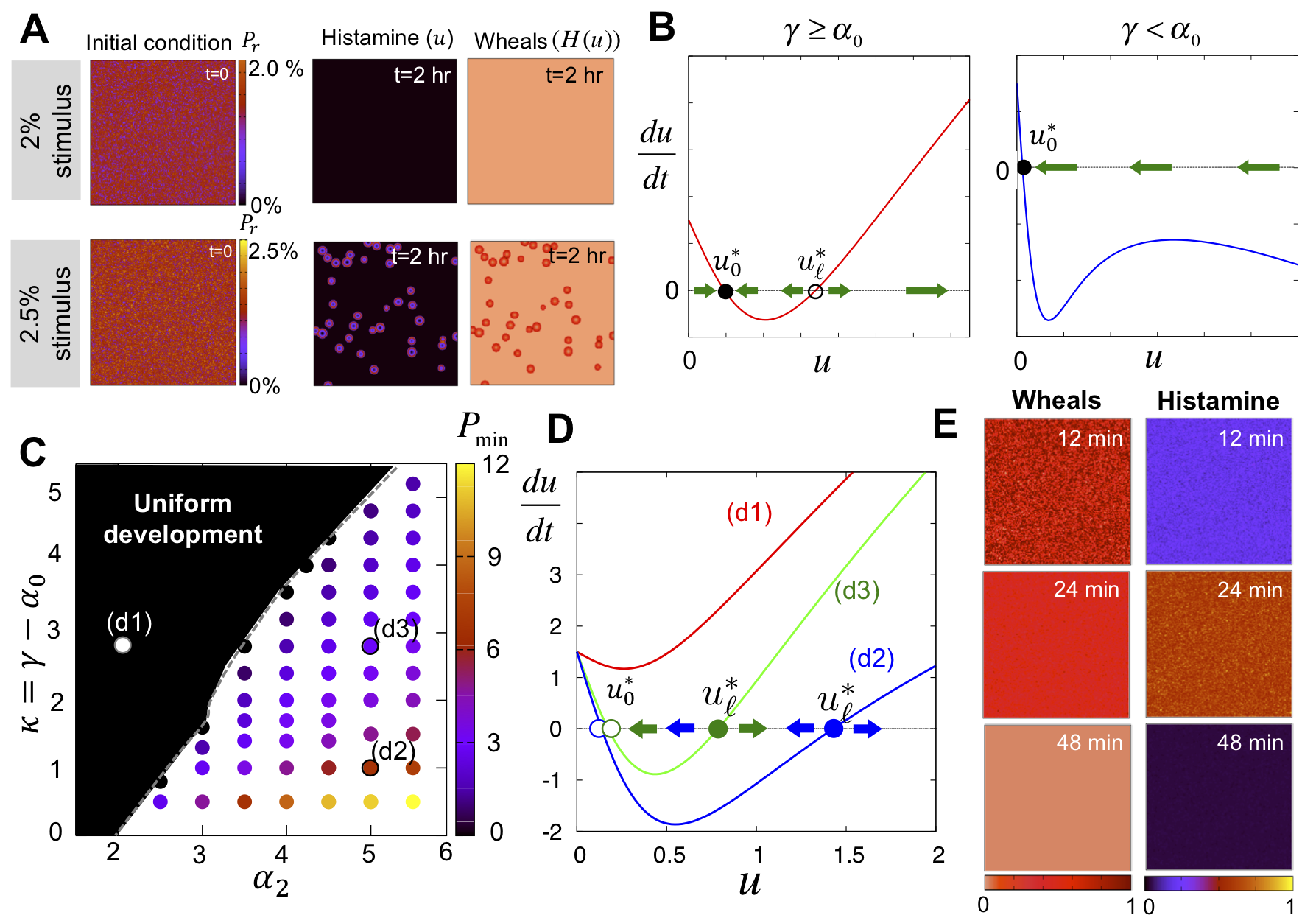

Supplement: S3 Fig — (A) Wheal emergence/non-emergence depending on Pr. The same parameters have been given for two simulations except for Pr. γ = 4.0, α1 = 0.4,α2 = 5.0,α0 = 0.7,μ = 1.5 are chosen as nondimensional parameters. The initial condition was given with s = 0. (B) The example graphs of du/dt (i.e. the case of Du = 0) for the size relation between histamine release rate (γ) and basal decay rate (α0). u0* and ul* are two positive equilibria. The white and black circles indicate a stable and unstable state in the given equilibrium, respectively. Arrows indicate the direction of histamine concentration around equilibria. (C) Parameter space for developing urticaria. In the black shaded region, uniform urticaria develops without a pattern. α1 = 0.4, μ = 1.5 are chosen in a nondimentional parameters. γ,α2,α0 are plotted with nondimensional scale. (d1) is (2.0, 3.0), (d2) is (5.0, 1.0), and (d3) is (5.0, 2.92). (D) The graphs of du/dt for each case, (d1), (d2) and (d3). (E) Time course of wheals and histamine distributions simulated by the parameter set of (d1) in C. (TIFF) [file pcbi.1007590.s004.tiff]

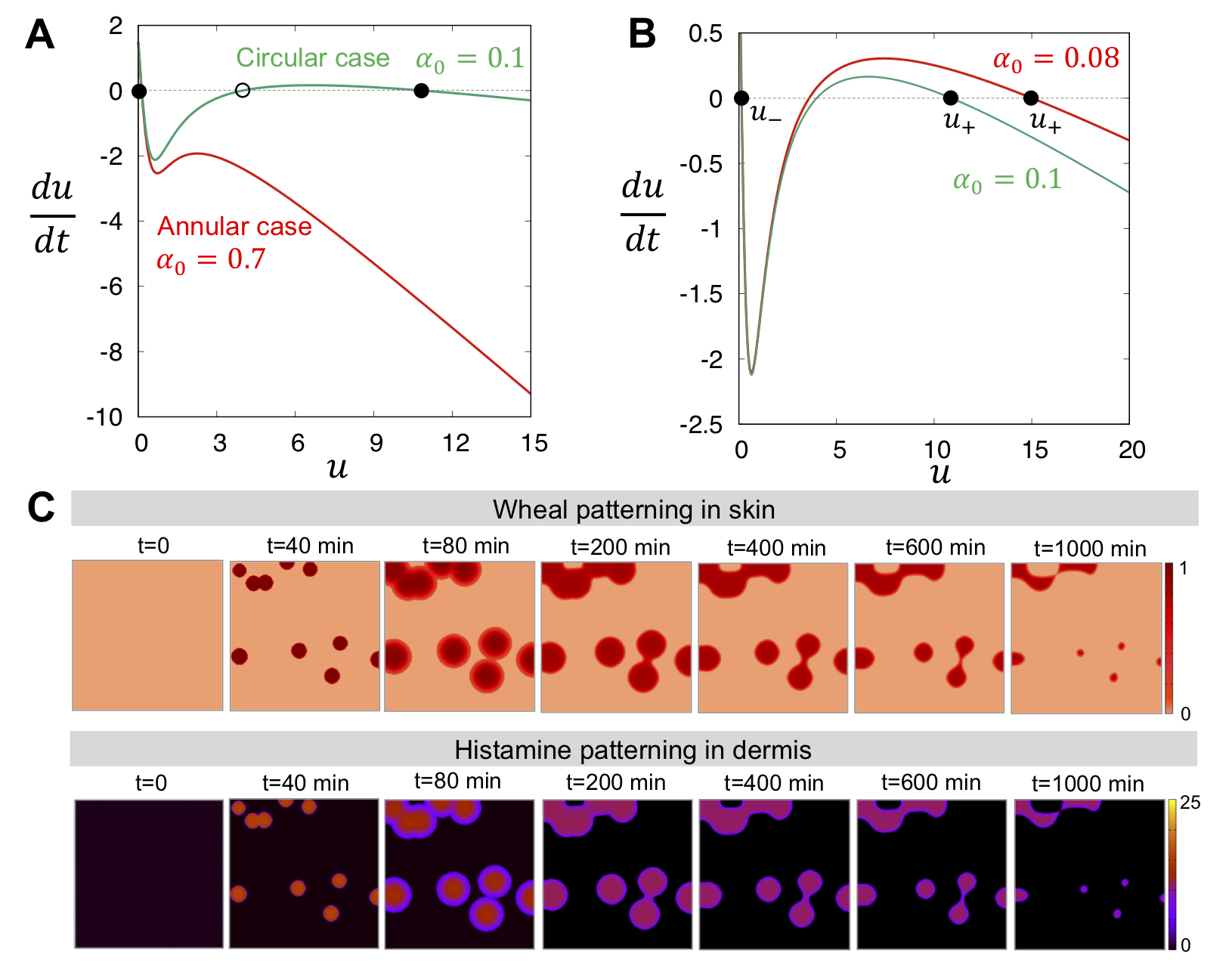

Supplement: S4 Fig — (A) The reaction terms of the equation (1.3) for annular patterns (red line) and circular patterns (green line). Shaded circle implies a stable equilibrium and blanked circle implies an unstable equilibrium. (B) The reaction term of the equation (1.3) for circular patterns with respect to the value of α0. u− and u+ are stable equilibria. (C) Temporal dynamics for the case of qα0 in Fig 3E. (TIFF) [file pcbi.1007590.s005.tiff]
