# Supplementary material for: A single reaction-diffusion equation for the multifarious eruptions of urticaria
Source: PLoS Comput Biol. 2020 Jan 15;16(1):e1007590. doi: 10.1371/journal.pcbi.1007590 (PMC6961880; doi:10.1371/journal.pcbi.1007590)
Supplement: S1 Table — (DOCX) [file pcbi.1007590.s006.docx]

Table S1. Fitting functions of $A_{w}, R_{w}, {dR}_{w}/dt$.
